# Describing symptoms using the Symptom Screening in Pediatrics Tool in hospitalized children with cancer and hematopoietic stem cell transplant recipients

**DOI:** 10.1002/cam4.1433

**Published:** 2018-03-23

**Authors:** Donna L. Johnston, Shannon Hyslop, Deborah Tomlinson, Christina Baggott, Paul Gibson, Andrea Orsey, David Dix, Vicky Price, Magimairajan Vanan, Carol Portwine, Susan Kuczynski, Brenda Spiegler, George A. Tomlinson, Laura Lee Dupuis, Lillian Sung

**Affiliations:** ^1^ Division of Hematology/Oncology Children's Hospital of Eastern Ontario 401 Smyth Road Ottawa Ontario K1H 8L1 Canada; ^2^ Program in Child Health Evaluative Sciences The Hospital for Sick Children Peter Gilgan Centre for Research and Learning 686 Bay Street Toronto Ontario M5G 0A4 Canada; ^3^ Pediatric Hematology/Oncology Stanford University Cancer Clinical Trials Office 800 Welch Road, MC 5757 Palo Alto California 94305; ^4^ Haematology/Oncology Department of Pediatrics London Health Sciences Centre 800 Commissioners Road East London Ontario N6A 5W9 Canada; ^5^ Division of Pediatric Hematology/Oncology Connecticut Children's Medical Center 282 Washington Street Hartford Connecticut 06106; ^6^ Division of Hematology/Oncology/BMT Department of Pediatrics BC Children's Hospital 4480 Oak Street Room B315 Vancouver British Columbia V6H 3V4 Canada; ^7^ Division of Hematology/Oncology Department of Pediatrics IWK Health Centre 5850/5980 University Avenue Halifax Nova Scotia B3K 6R8 Canada; ^8^ Departments of Pediatrics & Child Health and Biochemistry & Medical Genetics Pediatric Hematology/Oncology/BMT CancerCare Manitoba Research Institute in Oncology and Hematology University of Manitoba 675 McDermot Avenue Winnipeg Manitoba R3E 0V9 Canada; ^9^ Division of Haematology/Oncology McMaster Children's Hospital Health Sciences Centre 1280 Main Street West Hamilton Ontario L8S 4K1 Canada; ^10^ Ontario Parents Advocating for Children with Cancer (OPACC) 99 Citation Drive Toronto Ontario M2K 1S9 Canada; ^11^ Department of Psychology The Hospital for Sick Children 555 University Avenue Toronto Ontario M5G 1X8 Canada; ^12^ Department of Medicine Toronto General Hospital 200 Elizabeth Street Toronto Ontario M5G 2C4 Canada; ^13^ Department of Pharmacy The Hospital for Sick Children 555 University Avenue Toronto Ontario M5G 1X8 Canada; ^14^ Division of Haematology/Oncology The Hospital for Sick Children 555 University Avenue Toronto Ontario M5G 1X8 Canada

**Keywords:** Children, hematopoietic stem cell transplantation, oncology, symptom screening

## Abstract

Objectives were to describe any bothersome symptom and severely bothersome symptoms in inpatient children with cancer and hematopoietic stem cell transplant (HSCT) recipients. We included children 8–18 years of age with cancer or HSCT recipients who were receiving active treatment for cancer, admitted to hospital, and expected to be in hospital 3 days later. We administered the self‐report Symptom Screening in Pediatrics Tool (SSPedi). We described those who identified any degree of symptom bother (at least “a little”) and those who rated the degree of bother as severe (“a lot” or “extremely”). Factors associated with severe symptoms and total SSPedi scores were examined using multiple logistic and linear regression. Among the 302 patients, 298 (98.7%) reported having any bothersome symptom and 181 (59.9%) had at least one severely bothersome symptom. In multiple regression, older children were significantly more likely to have at least one severely bothersome symptom (15–18 and 11–14 years vs. 8–10 years; *P* = 0.008) and to have higher total SSPedi scores (*P* = 0.0003). Those with relapsed disease were more likely to have at least one severely bothersome symptom (odds ratio 2.1, 95% confidence interval 1.1–4.3; *P* = 0.037) and HSCT recipients were more likely to have higher symptom scores (*β *= 3.48, standard error = 1.6; *P* = 0.030). Almost all children receiving cancer therapies experience bothersome symptoms and 60% have at least one severely bothersome symptom. Older children experienced more severely bothersome symptoms and higher symptom scores. Future studies should follow children longitudinally to better understand the symptom trajectory and should institute interventions to manage symptoms.

## Introduction

Children with cancer and those who undergo hematopoietic stem cell transplantation (HSCT) have excellent survival outcomes related to intensive therapy 1. However, multiple studies have documented a high prevalence and intensity of symptoms during treatment 2, 3, 4. In response to an identified need for an appropriate symptom screening tool for children receiving cancer treatments 5, 6, 7, we developed the Symptom Screening in Pediatrics Tool (SSPedi). SSPedi is intended for children with cancer and pediatric HSCT recipients and includes 15 symptoms rated on a 5‐point Likert scale. Patients report how much each symptom bothered them yesterday or today 6, 8, 9. SSPedi also allows children to record additional bothersome symptoms not already listed as free text.

We recently validated SSPedi in 502 children with cancer and HSCT recipients. Reliability was excellent with intraclass correlation coefficients of 0.88 (95% confidence interval (CI) 0.82–0.92) for test–retest reliability, and 0.76 (95% CI 0.71–0.80) for inter‐rater reliability between guardians and children. SSPedi was responsive to change in global symptoms and all hypothesized relationships among measures for construct validation were observed. Using this data set then allowed the opportunity to describe the degree of symptom burden in the inpatient population.

Subsequently, our objectives were to describe any bothersome symptom and severely bothersome symptoms in inpatient children with cancer and HSCT recipients and to identify factors associated with these outcomes.

## Methods

This report was a subanalysis of data gathered as part of a primary study designed to evaluate the reliability, validity, and responsiveness of SSPedi 10. In the overall study, two groups of participants were enrolled namely an inpatient (target enrollment *n* = 300) and an outpatient (target enrollment *n* = 200) population. This report focuses on the inpatient cohort.

### Subjects

Respondents were English‐speaking children and adolescents 8–18 years of age with cancer or HSCT recipients. For this analysis, children receiving active treatment for cancer or undergoing HSCT, admitted to hospital, and expected to be in hospital or in clinic 3 days later were included. Exclusion criteria were illness severity, cognitive disability, or visual impairment that precluded completion of SSPedi as judged by the primary healthcare team. Participants could only be enrolled once.

### Procedures

Participants were enrolled from nine sites in Canada (*n* = 7) and the United States (*n* = 2) (Appendix 1). Research Ethics Board approval was obtained from all nine sites. Children and parents/guardians provided informed consent and children provided assent as appropriate. Demographic data were obtained from the respondents and from the medical records. Child respondents self‐reported SSPedi on an iPad on the date of enrollment and 3 days later (±1 day).

### Statistics

The total SSPedi score is calculated by summing each of the 15 items’ Likert scores, which consist of 0 = “not at all bothered,” 1 = “a little,” 2 = “medium,” 3 = “a lot,” and 4 = “extremely bothered” for a total score that ranges from 0 (none) to 60 (worst possible). We identified the number of children who rated any symptom as a score of 1 (a little) or higher, and the number of children who rated any symptom as a score of 3 (a lot) or 4 (extremely bothered).

We created two approaches to evaluate factors associated with symptoms at enrollment. First, we determined factors associated with having at least one severe symptom. For this approach, we conducted univariate and multiple logistic regression analyses. Second, we determined factors associated with the total symptom burden. To determine factors associated with the total SSPedi score, we conducted univariate and multiple linear regression. Only the initial SSPedi scores were considered for regression as multiple scores within a child would be correlated.

Factors evaluated were gender, age (8–10, 11–14 and 15–18 years), diagnosis (leukemia or lymphoma, solid tumor, brain tumor, and other), <3 months from diagnosis, metastatic disease, relapse, HSCT, reason for admission (chemotherapy or fever), parent received college or university education, and first language is English. Factors with a *P* value <0.1 in univariate analysis were evaluated in multiple regression. Statistical significance was considered a *P* value <0.05. Analyses were conducted using the SAS statistical program (SAS‐PC, version 9.4; SAS Institute Inc, Cary, NC).

## Results

Between November 11, 2014 and June 5, 2017, 649 children were assessed for eligibility. There were 64 who did not meet eligibility criteria and 66 who declined to participate, thus leaving 502 children who were enrolled in the study. Among the 502 participants, 302 were inpatients and were included in this analysis. The median age of participants was 13.2 years (range 8.0–18.5 years).

Among the 302 patients, 298 (98.7%) reported having at least one symptom of any degree of bother (≥ score of 1) and 181 (59.9%) had at least one symptom scored as “a lot bothered” or “extremely bothered” (score of 3 or 4) at enrollment. The median total SSPedi score at enrollment was 12 (interquartile range (IQR) 8–19). There were 282/302 (93.4%) children who completed SSPedi 3 days later and the median total SSPedi score was 9 (IQR 5–15). At enrollment, the most common symptoms of any degree of bother were feeling tired (*n* = 271, 89.7%), feeling more or less hungry than you usually do (*n* = 233, 77.2%) and changes in taste (*n* = 182, 60.3%). As previously described 10, the most common severely bothersome symptoms were feeling tired (*n* = 99, 32.8%), feeling more or less hungry than you usually do (*n* = 75, 24.8%), hurt or pain (other than headache) (*n* = 43, 14.2%), and changes in taste (*n* = 43, 14.2%).

Table 1 shows the characteristics of the entire cohort and stratified by the 181 participants who reported at least one severely bothersome symptom and the 121 who reported no severely bothersome symptoms. In total, 28.5% of the cohort were 8–10 years of age. The most common underlying diagnosis was leukemia or lymphoma (59.9%).

**Table 1:** Demographics of the study cohort

Characteristic	Total cohort (*N* = 302)	At least one severe symptom (*N* = 181)	No severe symptoms (*N* = 121)
Male gender	185 (61.3%)	108 (59.7%)	77 (63.6%)
Median age in years			
8–10	86 (28.5%)	40 (22.1%)	46 (38.0%)
11–14	128 (42.4%)	81 (44.8%)	47 (38.8%)
15–18	88 (29.1%)	60 (33.2%)	28 (23.1%)
Diagnosis			
Leukemia/Lymphoma	181 (59.9%)	100 (55.3%)	81 (66.9%)
Solid tumor	99 (32.8%)	65 (35.9%)	34 (28.1%)
Brain tumor	14 (4.6%)	12 (6.6%)	2 (1.7%)
Other	8 (2.7%)	4 (2.2%)	4 (3.3%)
Less than 3 months from diagnosis	147 (48.7%)	89 (49.2%)	58 (47.9%)
Metastatic disease	78 (25.8%)	46 (25.4%)	32 (26.4)
Relapse	52 (17.2%)	37 (20.4%)	15 (12.4%)
Stem cell transplantation	32 (10.6%)	23 (12.7%)	9 (7.4%)
Reason for admission: chemotherapy	237 (78.5%)	137 (75.7%)	100 (82.6%)
Reason for admission: fever	20 (6.6%)	14 (7.7%)	6 (5.0%)
Parent college or university	147 (48.7%)	80 (44.2%)	67 (55.4%)
First language English	253 (83.8%)	157 (86.7%)	96 (79.3%)

Table 2 illustrates the relationship between characteristics and having at least one severely bothersome symptom. Factors evaluated in multiple logistic regression were age at assessment, diagnosis group, relapse, parent completed college or university, and child's first language being English. In multiple regression analysis, older age category was significantly associated with having at least one severely bothersome symptom (*P* = 0.008); odds ratio (OR) for those who were 15–18 years of age was 2.5 (95% CI: 1.3–4.7) when compared to 8–10 years of age. Those with relapsed disease were also more likely to have at least one severely bothersome symptom (OR 2.1, 95% CI: 1.1–4.3; *P* = 0.037).

**Table 2:** Factors associated with severe symptoms

Characteristic	Univariate			Multiple		
	Odds Ratio	95% CI	*P* value	Odds Ratio	95% CI	*P* value
Male gender	0.8	0.5–1.4	0.488	NA		
Median age in years						
8–10	REF		0.009	REF		0.008
11–14	2.0	1.1–3.5		2.2	1.2–4.0	
15–18	2.5	1.3–4.6		2.5	1.3–4.7	
Diagnosis						
Leukemia/Lymphoma	0.6	0.4–1.1	0.086	0.6	0.4–1.1	0.112
Solid tumor	REF			REF		
Brain tumor	3.1	0.8–20.9		2.9	0.7–19.8	
Other	0.5	0.1–2.3		0.6	0.1–3.1	
Less than 3 months from diagnosis	1.1	0.7–1.7	0.833	NA		
Metastatic disease	0.9	0.6–1.6	0.841	NA		
Relapse	1.8	1.0–3.6	0.072	2.1	1.1–4.3	0.037
Stem cell transplantation	1.8	0.8–4.3	0.150	NA		
Reason for admission: chemotherapy	0.7	0.4–1.2	0.151	NA		
Reason for admission: fever	1.6	0.6–4.6	0.346	NA		
Parent college or university	0.6	0.4–1.0	0.058	0.6	0.4–1.0	0.057
First language English	1.7	0.9–3.2	0.090	1.8	0.9–3.4	0.095

NA, not applicable as not tested in multiple regression.

Table 3 shows the results of multiple linear regression when these same factors were evaluated against the total SSPedi score. In this analysis, male gender, age category, and HSCT were included in the multiple regression analysis. Again, older child age was associated with higher symptom scores (*P* = 0.0003); those who were 15–18 years of age had, on average, a 5.1 point increased total SSPedi score (standard error = 1.29) when compared to children 8–10 years. HSCT recipients also had higher symptoms scores (*P* = 0.030). The median total SSPedi score among the 32 HSCT recipients was 17 (IQR 8–25).

**Table 3:** Factors associated with higher SSPedi scores

Characteristic	Univariate			Multiple		
	*β*	SE	*P* value	*β*	SE	*P* value
Male gender	−2.00	1.03	0.053	−1.70	1.01	0.094
Median age in years						
8–10	REF		0.0005	REF		0.0003
11–14	3.77	1.20		3.68	1.19	
15–18	4.85	1.30		5.09	1.29	
Diagnosis			0.487	NA		
Leukemia/Lymphoma	−0.55	1.10				
Solid tumor	REF					
Brain tumor	3.11	2.51				
Other	−1.27	3.23				
Less than 3 months from diagnosis	1.54	1.01	0.127	NA		
Metastatic disease	−1.67	1.15	0.147	NA		
Relapse	0.16	1.33	0.905	NA		
Stem cell transplantation	3.05	1.63	0.062	3.48	1.60	0.030
Reason for admission: chemotherapy	−0.72	1.23	0.560	NA		
Reason for admission: fever	1.85	2.03	0.364	NA		
Parent college or university	−1.43	1.01	0.157	NA		
First language English	0.57	1.37	0.675	NA		

NA, not applicable as not tested in multiple regression.

## Discussion

We made several important observations in this early evaluation of symptoms using SSPedi. First, we found that the burden of bothersome symptoms is extremely high in this population. Virtually all inpatient children experienced at least one bothersome symptom and 60% experienced at least one severely bothersome symptom. Second, older children and those with relapsed disease were more likely to have at least one severely bothersome symptom and older children and HSCT recipients were more likely to have higher symptom bother scores. Interestingly, diagnosis was not associated with differences in symptoms experienced.

We found the degree of bothersome symptoms to be very high in an inpatient population, which is consistent with other studies that identified inpatient children with cancer as having more symptoms compared to outpatients 11, 12. It is important to emphasize that given the research nature of our study, parents and providers may have been reluctant to burden the most symptomatic children with a clinical study and thus, it is possible that our results are an underestimation of symptoms experienced. In contrast to our findings, one study that evaluated inpatient children for five consecutive days concluded that on the majority of hospitalization days, participants reported no overall symptoms or mild overall symptoms 3. It is difficult to directly compare these results since that study used the Memorial Symptom Assessment Scale (MSAS) Pediatric 10–18 scale, which assesses 31 symptoms and asks about three dimensions (frequency, severity, and distress) for 23 symptoms and two dimensions (severity and distress) for eight symptoms. The different nature of the scales may explain the divergence in results.

We identified older age as a risk factor for both experiencing at least one severely bothersome symptoms and for higher total symptom scores. These results are consistent with a study using the proxy MSAS 10–18, which similarly found that parents of adolescents reported more symptoms than parents of children 0–3 years of age 4. Also similar to our findings, they failed to find an association between symptom burden and underlying diagnosis.

In this analysis, we focused on general symptom burden by evaluating the proportion of children with any bothersome symptom, the proportion with at least one severely bothersome symptom and the total burden of symptom bother as measured using the SSPedi total scores. Future work should also evaluate each symptom separately to gain insight into how different symptoms vary in prevalence and associated risk factors.

An important question is how SSPedi could be incorporated into the routine clinical care of pediatric patients and its potential impact on outcomes. We have shown that children receiving cancer treatments have a high degree of bothersome symptoms. We hypothesize that routine symptom screening would highlight bothersome symptoms for healthcare providers and would lead to the earlier and more consistent application of interventions for symptom control. The ideal frequency of symptom screening is unknown and will likely differ between inpatients and outpatients. We also believe that if applied on a routine basis, SSPedi scores could become a metric of quality of care and could inform the effectiveness of different strategies for symptom prevention.

A strength of this study is that we measured symptoms from the child's perspective using a validated tool. Another strength is that we enrolled children from multiple sites, which improves the generalizability of our finding. However, our results should be interpreted in light of study limitations. One of the biggest limitations of this analysis is that it is cross‐sectional. Future studies will need to follow children longitudinally over time to better understand the symptom trajectory. Second, we assessed inpatients only; outpatients likely have a different pattern of symptom burden. Third, we only enrolled English‐speaking children.

In conclusion, almost all children receiving cancer therapies experience bothersome symptoms and 60% experience at least one severely bothersome symptom during admission to hospital. Older children are more likely to have severely bothersome symptoms and to have higher symptom scores. Future studies should follow children longitudinally to better understand the symptom trajectory and should institute interventions to prevent and treat symptoms.

## Conflict of Interest

The authors have declared no conflict of interests.

## Appendix 1 Participating sites.

**Table:**

Site	Location	Principal Investigator(s)
BC Children's Hospital	Vancouver, Canada	David Dix
CancerCare Manitoba	Winnipeg, Canada	Magimairajan Vanan
Children's Hospital, London Health Sciences Centre	London, Canada	Paul Gibson
Children's Hospital of Eastern Ontario	Ottawa, Canada	Donna Johnston
Connecticut Children's Medical Center	Hartford, United States	Andrea Orsey
IWK Health Centre	Halifax, Canada	Vicky Price
McMaster Children's Hospital	Hamilton, Canada	Carol Portwine
Stanford University School of Medicine	Palo Alto, United States	Christina Baggott
The Hospital for Sick Children	Toronto, Canada	Lillian Sung, Lee Dupuis
